# Signal encoding in magnetic particle imaging: properties of the system function

**DOI:** 10.1186/1471-2342-9-4

**Published:** 2009-04-01

**Authors:** Jürgen Rahmer, Jürgen Weizenecker, Bernhard Gleich, Jörn Borgert

**Affiliations:** 1 Philips Research Europe – Hamburg, Röntgenstraße 24-26, 22335 Hamburg, Germany

## Abstract

**Background:**

Magnetic particle imaging (MPI) is a new tomographic imaging technique capable of imaging magnetic tracer material at high temporal and spatial resolution. Image reconstruction requires solving a system of linear equations, which is characterized by a "system function" that establishes the relation between spatial tracer position and frequency response. This paper for the first time reports on the structure and properties of the MPI system function.

**Methods:**

An analytical derivation of the 1D MPI system function exhibits its explicit dependence on encoding field parameters and tracer properties. Simulations are used to derive properties of the 2D and 3D system function.

**Results:**

It is found that for ideal tracer particles in a harmonic excitation field and constant selection field gradient, the 1D system function can be represented by Chebyshev polynomials of the second kind. Exact 1D image reconstruction can thus be performed using the Chebyshev transform. More realistic particle magnetization curves can be treated as a convolution of the derivative of the magnetization curve with the Chebyshev functions. For 2D and 3D imaging, it is found that Lissajous excitation trajectories lead to system functions that are closely related to tensor products of Chebyshev functions.

**Conclusion:**

Since to date, the MPI system function has to be measured in time-consuming calibration scans, the additional information derived here can be used to reduce the amount of information to be acquired experimentally and can hence speed up system function acquisition. Furthermore, redundancies found in the system function can be removed to arrive at sparser representations that reduce memory load and allow faster image reconstruction.

## Background

"Magnetic Particle Imaging" (MPI) is a method for imaging distributions of magnetic nano-particles which has been introduced recently [1]. For generating a detectable particle signal, the method exploits the non-linear magnetization response of ferromagnetic particles to an externally applied oscillating magnetic *drive field*. The magnetization response induces a voltage in receive coils, which constitutes the MPI signal.

**Figure 1:**
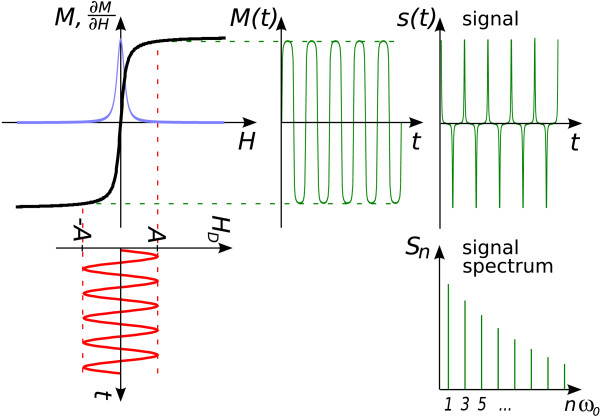
**Basic MPI Principle**. Basic MPI principle [1]. The drive field *H*_*D*_(*t*) generates a particle response *M*(*t*) that induces a voltage in the receive coils. The time-dependent voltage is measured and constitutes the raw signal *s*(*t*) ∝ d*M*(*t*)/d*t*. Due to the non-linear magnetization curve, the signal spectrum *S*_*n *_contains higher harmonics of the drive frequency *ω*_0_, which are used for particle detection and imaging. For reference, the derivative of the magnetization curve *∂M*/*∂H *is also shown (blue curve).

As shown in figure 1, its spectrum contains higher harmonics of the drive frequency, which represent the fingerprint of the particles. Spatial localization is achieved by superimposing an inhomogeneous, static magnetic *selection field*, that limits the particle response to a small region, also called *field free point *(FFP). The effect of the drive field is to move the FFP in space. Using orthogonal sets of drive field coils, fast spatial coverage with a 2D or 3D trajectory of the FFP can be achieved, allowing real-time tomographic imaging of particle distributions [2].

Since the particles' magnetic moment is about eight orders of magnitude larger than the proton magnetic moment used in MRI, MPI achieves high sensitivity [3]. This makes it a promising candidate for dynamic medical imaging applications, e.g., blood flow imaging in coronary arteries. Feasibility of 3D real-time visualization of blood flow through the vascular system of a living mouse has been demonstrated recently [4].

For MPI, in contrast to established imaging modalities like MRI and CT [5], no simple mathematical transform has yet been identified to reconstruct images from the acquired data. Therefore, MPI image reconstruction requires knowledge of a "system function" describing the system response to a given spatial distribution of particles, i.e., mapping particle position to frequency response. To solve the reconstruction problem, the system function has to be inverted, usually requiring some regularization scheme [3]. To date, the system function is determined experimentally by measuring the magnetization response of a point-like sample at a large number of spatial positions corresponding to the number of image pixels or voxels [1]. This calibration procedure requires very long acquisition times and furthermore provides a system function that is contaminated with noise. Due to the large size of the system function matrix, solving the inverse reconstruction problem is also quite time-consuming and claims large amounts of computer memory.

From a theoretical understanding of the signal encoding process one expects to gain insight into the structure of the system function. This knowledge can be used to speed up the system function acquisition or to even simulate parts or all of the system function. Information about the matrix structure can furthermore help to find more compact system function representations, helping to reduce memory requirements and speed up reconstruction. Finally, identification of a mathematical transform leading from the data to the image would greatly simply the reconstruction process.

## Methods

### Signal Generation

The basic principle of signal generation in MPI relies on the non-linear magnetization response ***M***(***H***) of ferromagnetic particles to an applied magnetic field ***H***, cf. figure 1. An oscillating *drive field ****H***_D_(*t*) of sufficient amplitude leads to a magnetization response ***M***(*t*) of the particles, which has a different spectrum of higher harmonics than the drive field. If, for instance, a harmonic drive field is used, the drive field spectrum only contains the base frequency, whereas the particle response also contains multiples thereof. The information contained in these higher harmonics is used for MPI. Experimentally, the time-dependent change in particle magnetization is measured via the induced voltage in receive coils. Assuming a single receive coil with sensitivity ***σ***_*r*_(***r***) at spatial position ***r***, the changing magnetization induces a voltage(1)

according to Faraday's law. *μ*_0 _is the magnetic permeability of vacuum. The receive coil sensitivity ***σ***_*r*_(***r***) = ***H***_*r*_(***r***)/*I*_0 _derives from the field ***H***_*r*_(***r***) the coil would produce if driven with a unit current *I*_0 _[5].

In the following, the sensitivity of the receive coil is approximated to be homogeneous over the region of interest, i.e., ***σ***_*r*_(***r***) is constant. If *M*_*x*_(***r***, *t*) is the magnetization component picked up by a receive coil in *x *direction, i.e., having the sensitivity ***σ***_*r *_= (*σ*_*x*_, 0, 0)^*T*^, the detected signal can be written as(2)

Neglecting constant factors, we introduce the notation *s*(***r***, *t*) for signal generated by a point-like distribution of particles at position ***r***. If the particle distribution is approximated by a *δ *distribution, the volume integral vanishes and the particle magnetization *M*_*x*_(***r***, *t*) is determined by the local field ***H***(***r***, *t*). For the moment, the field is assumed to have only one spatial component *H*_*x*_(***r***, *t*), which is pointing in receive-coil direction *x*. The signal can then be written as(3)

**Figure 2:**
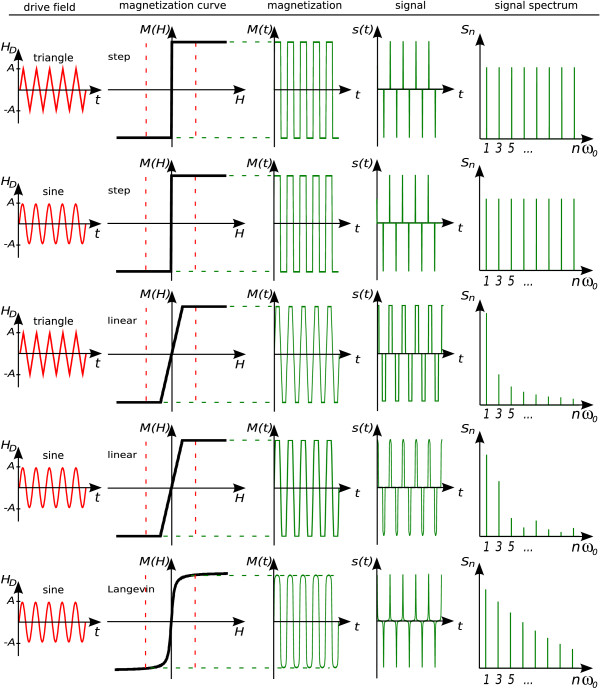
**Particle Magnetization Response**. Particle magnetization response *M*(*t*), acquired time signal *s*(*t*), and magnitude spectral components *S*_*n *_for different drive fields and particle magnetization curves.

Since this equation holds for a general 1D setup where the field is aligned with the direction of the acquired magnetization component, the subscript *x *has been omitted. Equation 3 shows that high signal results from the combination of a steep magnetization curve with rapid field variations. Fourier expansion of the periodic signal *s*(*t*) generated by applying a homogeneous drive field *H*(***r***, *t*) = ***H***_D_(*t*) yields the signal spectrum *S*_*n*_, as shown in figure 1. Intensity and weight of higher harmonics in the spectrum are related to the shape of the magnetization curve *M*(*H*), and to the waveform and amplitude of the drive field *H*_D_(*t*). To illustrate their influence on the spectrum, a number of representative cases are shown in figure 2.

The step function relates to an immediate particle response and creates a spectrum that is rich in high harmonics. The spectral components have constant magnitude at odd multiples of the drive frequency. The even harmonics are missing due to the sine-type pattern of the time signal *s*(*t*). *The step function corresponds to an ideal particle response *and represents the limiting case for the achievable weight of higher harmonics. For this magnetization curve, triangle and sine drive fields yield the same result.

If the particle response to the drive field is slowed down by introducing a linear range in the magnetization curve, the relative weight of higher harmonics is reduced. Thereby, the harmonic drive field performs better than the triangular excitation, since it sweeps faster over the linear range.

The last row in figure 2 shows a more realistic particle magnetization as given by the Langevin function [6]

(4) *M*(*ξ*) = *M*_0 _(coth *ξ *- 1/*ξ*),

where *M*_0 _is the saturation magnetization and *ξ *is the ratio between magnetic energy of a particle with magnetic moment *m *in an external field ***H***, and thermal energy given by the Boltzmann constant *k*_B _and temperature *T*:(5)

A higher magnetic moment results in a steeper magnetization curve and creates more higher harmonics for a given drive field amplitude. Alternatively, high harmonics can be generated from a shallow curve using faster field variations, e.g., induced by a higher drive field amplitude. It should be noted that MPI uses ferromagnetic particles to obtain a sufficiently steep magnetization curve [1]. For low concentrations, however, their mutual interactions can be neglected and they can be treated like a gas of paramagnetic particles with extremely large magnetic moment, a phenomenon also known as "super-paramagnetism" [7].

### 1D Spatial Encoding

To encode spatial information in the signal, a static magnetic gradient field ***H***_S_(***r***), also called *selection field*, is introduced. For 1D encoding, the selection field has a non-zero gradient only in *x *direction, *G*_*x *_= d*H*_S_/d*x*. If the gradient is non-zero over the complete field of view (FOV), the selection field is monotonically rising or falling, thus it can cross zero only in a single point, the above-mentioned FFP. In regions far away from the FFP, the particle magnetization is driven into saturation by the selection field.

Application of a spatially homogeneous and temporally periodic drive field *H*_D_(*t*) in addition to the selection field *H*_S_(***r***) corresponds to a periodic displacement of the FFP along the gradient direction. The particles experience a local field

(6) *H*(*x*, *t*) = *H*_S_(*x*) - *H*_D_(*t*).

The minus sign has been chosen to make later calculations more convenient. Since the FFP sweeps over each spatial position *x *at a different point in time, each position can be identified by its characteristic spectral response.

#### Harmonic Drive Field – Ideal Particles

**Figure 3:**
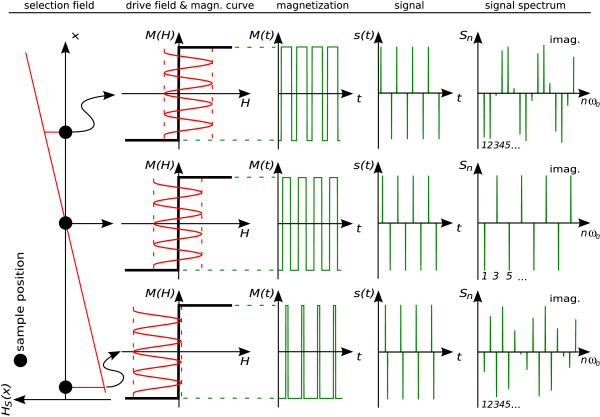
**Dependence of Magnetization Response on Selection Field Offset**. Relation between ideal particle response and selection field offset. A constant gradient *G *is assumed leading to a selection field *H*_S_(*x*) = *Gx*. The superposition of selection field *H*_S_(*x*) and harmonic drive field *H*_D_(*t*) = *A *cos *ω*_0_*t *generates a magnetization response *M*(*x*, *t*) which induces a voltage proportional to *s*(*x*, *t*) in the receive coil. Depending on position *x*, the signal spectrum has a characteristic pattern *S*_*n*_(*x*). Since the signal *s*(*t*) is real and odd, its Fourier components are purely imaginary.

**Figure 4:**
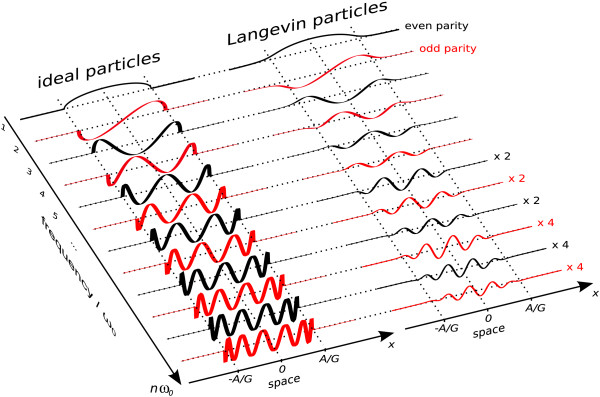
**Spatial Dependence of Spectral Signal Components**. Spatial dependence of spectral signal components for a harmonic drive field in combination with a constant gradient selection field. Left: ideal particles. Right: Langevin particles. For these more realistic particles, the spatial response functions extend beyond the range [-*A*/*G*; *A*/*G*] covered by the FFP motion.

Figure 3 shows spectra at three different spatial positions generated by ideal particles exposed to a selection field *H*_S _of constant gradient strength *G *and a harmonic drive field *H*_D _of frequency *ω*_0 _= 2*π*/*T *and amplitude *A*. A derivation of their functional form is given in appendix A.1 and A.2. For the *n*th harmonic, corresponding to the *n*th component of a Fourier series expansion, one finds the following dependence on particle position *x*:(7)

where the *U*_*n*_(*x*) represent Chebyshev polynomials of the second kind (42). The functions are defined in the range -1 <* Gx*/*A *< 1. A cosine drive field has been used instead of the sine drive field to arrive at a simpler expression. The spatial dependence for the first harmonics is plotted in the left part of figure 4. One finds an increasing number of oscillations with increasing frequency components *n*. This relates to the fact that Chebyshev polynomials form a complete orthogonal basis set (cf. appendix A.2), so that any particle distribution *C*(*x*) can be expanded into these functions. Successive frequency components have alternating spatial parity with respect to the center of the FOV (even/odd).

The *S*_*n*_(*x*) can be seen as a sensitivity map, describing the spatial sensitivity profile of each frequency component *n*. In an MPI experiment, a 1D particle distribution *C*(*x*) would generate spectral signal components(8)

Thus, the *S*_*n*_(*x*) represent the *system function *introduced by [3]. The system function not only describes the spatial signal dependence, but also contains information about the particles' magnetization curve and system parameters (e.g. drive field amplitude A and frequency *ω*_0 _= 2*π*/*T*, selection field gradient *G*).

Using (7), the spectral signal components (8) for ideal particles can be written as(9)

In this notation, the *V*_*n *_correspond to coefficients of a Chebyshev series, cf. equation (58). It follows that the particle concentration can be reconstructed by doing a Chebyshev transform of the measured *V*_*n*_, i.e., by evaluating the Chebyshev series(10)

Hence, for ideal particles under the influence of a harmonic drive field and a constant selection field gradient, reconstruction of the spatial particle distribution simply corresponds to calculating the sum over the measured harmonics *V*_*n *_weighted with Chebyshev polynomials of the second kind. In terms of the system function , the particle concentration can be written as(11)

#### Harmonic Drive Field – Langevin Particles

For more realistic particles as described by (4), the system function *S*_*n*_(*x*) is given by a spatial convolution between the derivative of the magnetization curve, *M'*(*H*_S_), and the Chebyshev components, as derived in A.2. Using equation (7) one can write:(12)

Depending on the steepness of *M*(*H*), the *S*_*n*_(*x*) will be a blurred version of the , extending slightly beyond the interval which is covered by the FFP motion and to which the  are confined. Thus, particles that are located slightly outside the range accessed by the FFP also generate signal. The right part of figure 4 displays components of the system function for particles following the Langevin magnetization curve in a constant selection field gradient.

In the measurement process according to (8), the FOV now refers to the range where the *S*_*n*_(*x*) are non-zero. A sufficiently steep magnetization curve can provide confinement to a region not much larger than the range covered by the FFP, i.e., -*A*/*G *<* x *<*A*/*G*.

Since the system function components cannot be sharper than the convolution kernel, an MPI experiment with Langevin particles will run into a resolution limit correlating with the width of *M'*(*x*). Since the derivative of the magnetization curve is a symmetric function *M'*(*x*) = *M'*(-*x*), one can use (12) to show that(13)(14)

where  corresponds to the expression in square brackets in (13). Since (14) corresponds to (9), reconstruction for the harmonic drive field is given by (11), i.e.,(15)

This means, that in the interval where the ideal particle system function  is defined, i.e., -*A*/*G *<* x *<*A*/*G*,  can be directly reconstructed. If the particle concentration *C*(*x*) is confined to the FOV,  is just the convolution of *C*(*x*) with *M'*(*x*):(16)

From this equation, one can infer that the resolution of the reconstructed image is limited by the width of *M'*(*x*). If the particle magnetization curve is known and the measurement provides sufficient SNR, deconvolution can be used to overcome this limitation. However, in practical applications with distributions of different particles sizes and magnetization curves, deconvolution may be difficult. Therefore, the following paragraph gives an estimation of the achievable spatial resolution without deconvolution. The derivative of the Langevin curve in equation (4) is(17)

which corresponds to the blue curve plotted in figure 1. The full width at half maximum (FWHM) of this curve can be determined numerically as Δ*ξ*_FWHM _≈ 4.16. If the particle magnetization *m *and the selection field gradient strength *G *are known, this can be translated to a spatial resolution using equation (5):(18)

The particle magnetization depends on particle diameter d according to the following relation [8]:(19)

**Figure 5:**
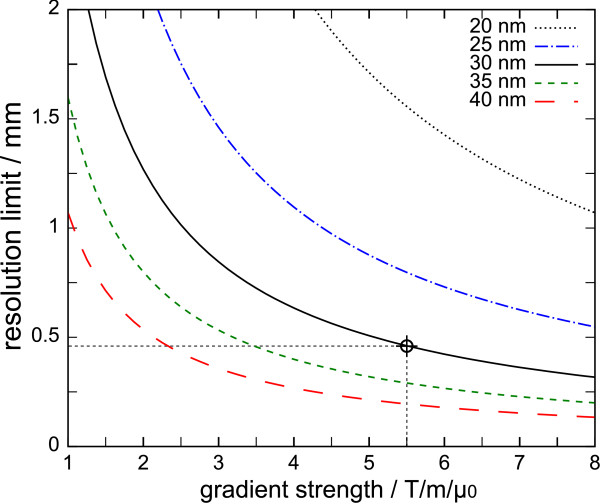
**Resolution Limit (without Deconvolution) for Different Particle Sizes as a Function of Gradient Strength**. Resolution Δ*x *achievable without deconvolution for different diameters *d *of magnetite particles (Fe_3_O_4_) as a function of gradient strength *G*. According to equations (18) and (19), the limit is proportional to *d*^-3 ^and *G*^-1^. The cross-hairs indicate a gradient/particle combination used in recent *in vivo *experiments [4].

Assuming magnetite particles (Fe_3_O_4_) with a saturation magnetization of *M*_S _= 0.6 T/*μ*_0_, the resolution limit Δ*x *imposed by the magnetization curve can be calculated. Figure 5 shows Δ*x *as a function of gradient strength for different particle diameters. The cross-hairs indicate the gradient strength of 5.5 T/m/*μ*_0 _and the dominating particle diameter of 30 nm used in real-time *in vivo *MPI [4]. This gradient/particle combination theoretically allows a resolution better than 0.5 mm. However, due to the wide distribution of particle sizes and the regularization applied in reconstruction [3] to mitigate limited SNR, the observed resolution was not better than Δ*x *≈ 1.5 mm.

#### Triangular Drive Field

An illustrative case is to use a triangular drive field instead of the harmonic field, cf. appendix A.3. The system function for ideal particles then has the form(20)

for an FFP motion covering the range 0 <* x *< 2*A*/*G*. Now, instead of the Chebyshev series, a Fourier series can be used to reconstruct a particle concentration(21)

The measured frequency components *V*_*n *_are proportional to components  in *k *space, which are related to the spatial distribution *C*(*x*) by Fourier transformation. In terms of the system function, (21) becomes(22)

For realistic particles, the system function has to be convolved with *M'*(*H*_S_). Since equations (12–14) derived for harmonic drive field excitation hold for the triangular system function as well, a modified/convolved concentration  can be reconstructed in the range 0 <* x *< 2*A*/*G*.

### Matrix Formulation

For MPI image reconstruction, the continuous spatial distribution of particles will be mapped to a grid, where each grid location represents a small spatial region. Furthermore, only a limited number *n *of frequency components is recorded. If the spatial positions are indexed with *m*, (8) becomes(23)

or, in vector/matrix formulation,

(24) ***v ***= ***Sc***.

Calculation of the concentration vector then basically corresponds to an inversion of matrix *S*:

(25) ***c ***=*** S***^-1^***v***.

This notation will be used for 2D or 3D imaging as well, which requires collapsing spatial indices into the single index *m*. Thus, concentration is always a vector, independent of spatial dimension.

Going back to the 1D case for a harmonic drive field, introduction of a scalar(26)

and a diagonal matrix(27)

allows derivation of the following identity by comparing (25) with (11):

(28) ***S***^-1 ^= *α***βS**^*T*^.

Thus, in the case of 1D imaging of ideal particles, the inverse matrix can simply be obtained by multiplication of the transpose with a scalar and a diagonal matrix.

Using only a limited number of frequency components corresponds to working with a truncated Chebyshev series. The Chebyshev truncation theorem then states that the error in approximating the real concentration distribution is bounded by the sum of the absolute values of the neglected coefficients. More importantly, for reasonably smooth distributions, the error is on the order of the last retained Chebyshev coefficient [9].

### 2D and 3D Spatial Encoding

#### 1D Drive Field

A first step towards describing 2D and 3D imaging is to look at the 3D system function of particles in a 3D selection field ***H***_S_(***r***) combined with a 1D drive field ***H***_D_(*t*). Using a harmonic drive field and choosing a Maxwell coil setup to create a selection field as described in equation (68), the total field can be approximated by(29)

The system function can be written as a convolution over the *z *component of the selection field (cf. (65))(30)

In this vector, each component refers to the signal induced by the respective *x*/*y*/*z *magnetization component. Detection of these components requires three orthogonal (sets of) receive coils. For ideal particles (cf. (66)), the explicit spatial dependence becomes (cf. (70))(31)

where the asterisk denotes convolution over the *z *component, i.e., the direction of the FFP motion resulting from the drive field. Thus, an expression describing the 3D spatial dependence of the respective magnetization component is convolved in drive field direction with the set of 1D Chebyshev functions.

**Figure 6:**
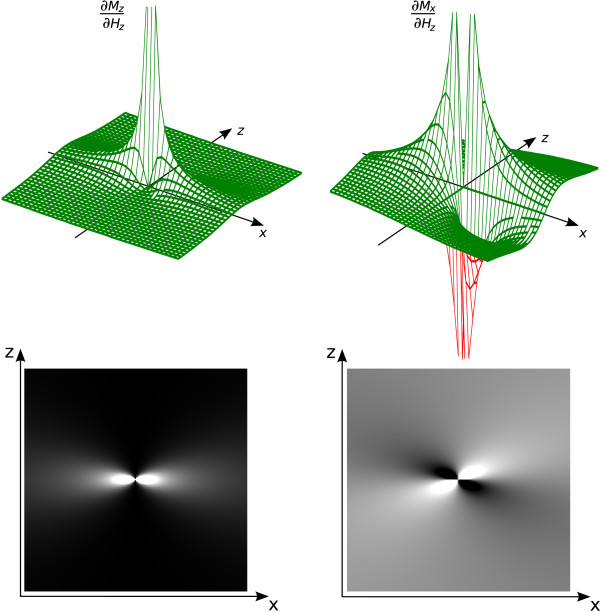
**Convolution Kernel Resulting from Magnetization Derivative**. Derivative of ideal particle magnetization with respect to the *H*_*z *_field component. The derivatives of the *M*_*z *_(left) and *M*_*x *_component (right) are shown. Top and bottom row show a 3D view and gray-scale plot of the *xz *plane, respectively. The displayed functions represent the convolution kernel applied to the basis set in drive field direction, cf. equation (31).

The shape of the convolution kernel is determined by *∂****M***/*∂****H***_*z*_, which describes how the magnetization responds to the drive field change. For ideal particles, it is singular at the origin. Figure 6 shows the *xz *plane of the 3D kernel for the signal components detected in *z *and *x *direction, *S*_*n*,*z*_(***r***) and *S*_*n*,*x*_(***r***), respectively. Along the center line in drive field direction, the kernel for the *M*_*z *_magnetization corresponds to the delta distribution, just as in the 1D situation (48). With increasing distance from the center line, the kernel broadens and its amplitude decreases rapidly. For *M*_*x*_, and for symmetry reasons also for *M*_*y*_, the kernel is zero on the symmetry axes. It has high amplitude close to the singularity at the origin.

**Figure 7:**
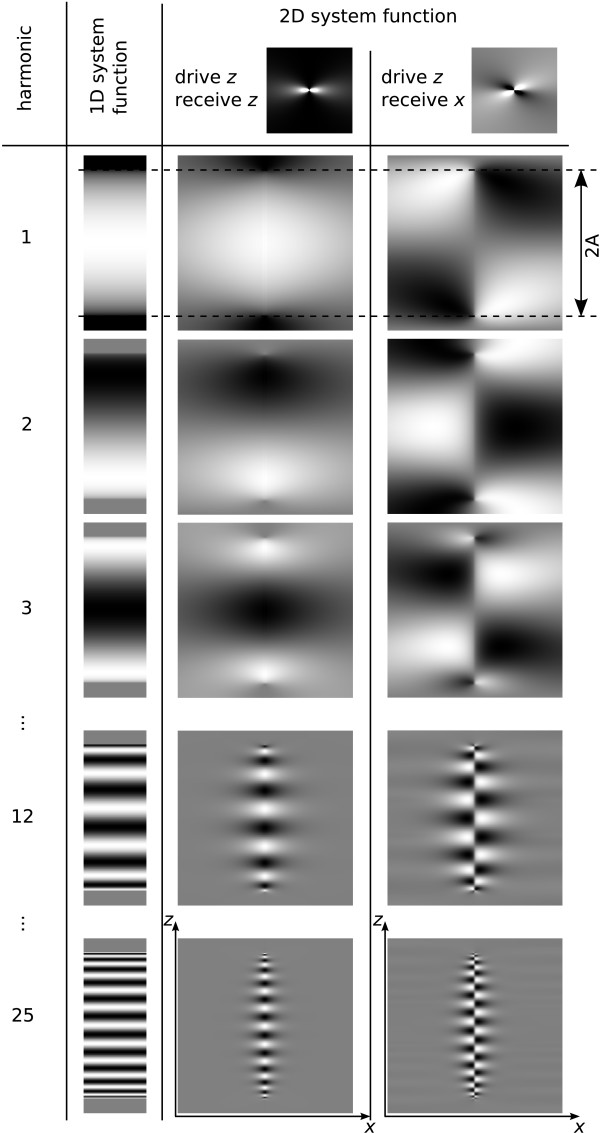
**2D Ideal Particle System Function for 1D FFP Motion**. Ideal particle system function at different harmonics for 1D FFP motion along the center line in *z *direction. The column '1D system function' displays the Chebyshev functions plotted in the left part of figure 4. The 2D system function is obtained from a convolution of the Chebyshev functions with the kernels shown in the top row for signal reception in *z *and *x *direction, respectively. The dashed lines indicate the edges of the range covered by the FFP motion.

To form the 3D ideal particle system function, the 3D kernel is convolved along the drive field direction with the 1D Chebyshev polynomials (31). Figure 7 shows central 2D slices extracted from selected harmonics for the above case of 1D drive field motion in *z *direction. Directly on the line covered by the FFP trajectory, the system function is given by Chebyshev polynomials and therefore can encode an arbitrary particle distribution, as discussed for the 1D situation. With increasing distance to the center line, the convolution kernel has an increasing blurring effect, so that finer structures of the higher Chebyshev polynomials are averaged to zero. Therefore, the signal in higher system function components condenses to the line of the FFP trajectory (cf. figure 7, harmonic 12 and 25), where the blurring effect is low. This can be explained by the fact that only in the close vicinity of the FFP, the field change is rapid enough to stimulate a particle response that generates high frequency components.

In an MPI experiment, it can be useful to exploit symmetries in the system function to partially synthesize the system function and thus speed up its acquisition and reduce memory requirements. From the 3D response to the 1D FFP motion, two basic rules can be derived for the parity of the system function in the spatial direction indexed with *i *∈ {*x*, *y*, *z*}.

1. The "base" parity is given by the parity of the convolution kernel shown in figure 6. It is even, if the receive direction *j *∈ {*x*, *y*, *z*} is aligned with the drive direction *k *∈ {*x*, *y*, *z*}. This corresponds to the magnetization derivative component *∂M*_*j*_*∂H*_*k *_for *j *= *k*. Otherwise kernel parity is odd:(32)

2. If the spatial direction of interest is a drive field direction, i.e., *i *= *k*, then parity alternates between successive harmonics *h *of that drive field component:(33)

The reason is the alternating parity of the Chebyshev polynomials in the 1D system function.

Parity observed for harmonic *h *in a spatial direction *i *then is *p*_*i*,*j*,*k*,*h *_= *p*_Cheb_·*p*_kernel_.

#### 2D and 3D Drive Field

**Figure 8:**
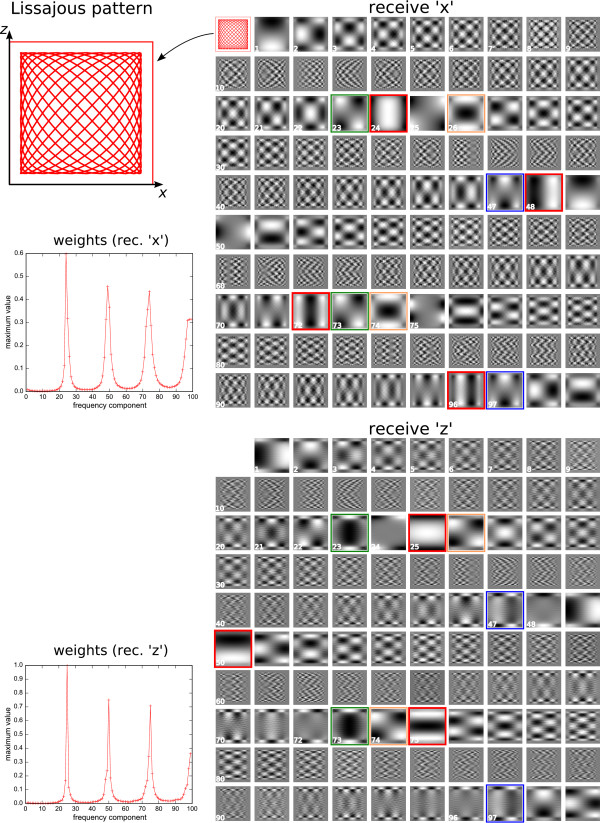
**2D Ideal Particle System Function for 2D Lissajous FFP Motion**. Successive frequency components of the ideal particle system function for 2D Lissajous FFP motion with an *x*/*z *frequency ratio of 24/25. The first 99 frequency components are shown for the two receive orientations. The first upper left rectangle displays the Lissajous pattern. Multiples of the drive frequencies are indicated by red frames. Their shape closely resembles the 1D system function shown in figure 7. Green, blue, and orange frames indicate components with identical spatial pattern. The two graphs at the left show the maximum intensity values encountered in the respective system function components.

As displayed in figure 7, the spatial pattern of the particle response for higher harmonics is confined to a narrow region close to the line of the FFP motion. Therefore, to induce a particle response with high harmonics extending over the whole 2D plane, a lateral displacement of the FFP is necessary. This is achieved by adding a second drive field which displaces the FFP in *x *direction. For 3D encoding, a third drive field for FFP displacement in *y *direction is necessary. Figure 7 furthermore shows that high resolution is obtained only in the close vicinity of the FFP trajectory line. Thus, the 2D or 3D trajectory should be sufficiently dense to achieve homogeneous resolution over the imaging plane or volume. For a simple implementation, one can choose harmonic drive fields with slight frequency differences in the orthogonal spatial directions, causing the FFP motion to follow a 2D or 3D Lissajous pattern. In the following, a 2D system function requiring two drive frequencies is investigated. The treatment of a 3D system function using three drive frequencies would be analogous. Figure 8 displays a 2D Lissajous pattern generated by the superposition of two orthogonal harmonic drive fields with frequency ratio *ω*_*x*_/*ω*_*z *_= 24/25:(34)

Using a 3D selection field according to (29), the doubled selection field gradient in *z *direction requires *A*_*z *_= 2*A*_*x *_to cover a quadratic FOV with the FFP motion. Figure 8 displays the first components of a simulated 2D system function for ideal particles exposed to the superposition of the 2D Lissajous drive field and the 3D selection field. Each receive direction has its own set of system functions, denoted by 'receive *x' *and 'receive *z'*. Components corresponding to higher harmonics of the respective drive frequency are indicated by red frames. On the *x *channel, they have a spacing of 24 components. In the spatial *x *direction, they closely resemble the 1D Chebyshev series, while in *z *direction, they show no spatial variation. On the *z *channel, harmonics of the drive frequency exhibit a spacing of 25 components with a spatial pattern that is basically rotated by 90 degrees with the respect to the *x *components.

While components corresponding to harmonics of the drive field frequencies only allow 1D encoding in the respective drive field direction, components arising from a mixture of both drive frequencies provide spatial variation in both directions at the same time. For instance, moving to the left from the first *x *drive-field harmonic on the *x *channel (component 24) corresponds to mix frequencies *mω*_*x *_+ *n*(*ω*_*x *_- *ω*_*z*_) with increasing integer *n *and *m *= 1. For larger *m*, one starts at a higher harmonic *m*. Moving to the right corresponds to negative *n*. Thus, pure drive field harmonics and their vicinity relate to low mixing orders, while increasing distance goes along with larger *n *and higher mixing orders.

It should be noted that the system function component observed for *mω*_*x *_+ *n*(*ω*_*x *_- *ω*_*z*_) appears a second time at frequency *mω*_*x *_+ *n*(*ω*_*x *_+ *ω*_*z*_). Thus every component corresponding to frequency mixes appears twice. Examples are components 23 and 73 (*m *= 1, *n *= 1, green frames) or 47 and 97 (*m *= 2, *n *= 1, blue frames), but also 26 and 74 (*m *= 1, *n *= -2, orange frames) on the *x *channel.

Figure 8 also plots the maximum intensities (weights) of the generated system function components. Highest intensities are found in pure multiples of the drive frequencies, however with a decrease towards higher frequencies. Components corresponding to mix frequencies have much lower intensity than pure harmonics. If the system function is acquired experimentally, these components will be the first to fall below the noise level. Thus, low SNR in the system function acquisition will reduce the achievable resolution.

**Figure 9:**
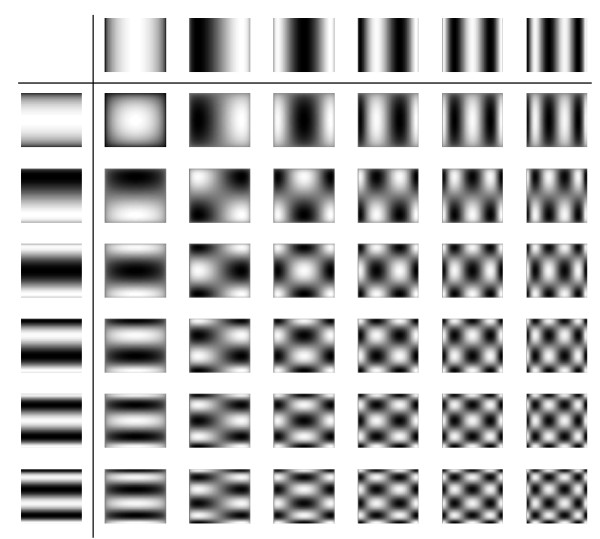
**Pictorial Table of 2D Chebyshev Functions**. Pictorial table of 2D Chebyshev functions. Top row and left column display the 1D Chebyshev functions, from which the 2D functions are derived by tensor multiplication.

**Figure 10:**
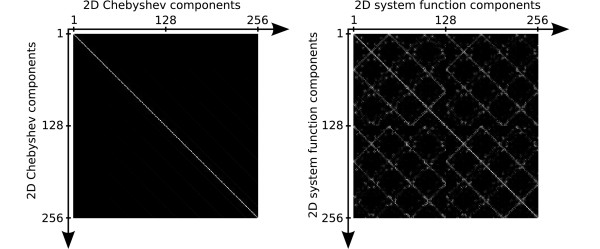
**Orthogonality Plots**. Orthogonality plot for the first 256 basis set components. Left: 2D Chebyshev basis set. Right: 2D MPI ideal particle system function for Lissajous FFP motion with frequency ratio 24/25. System function matrix rows were normalized.

The higher the order of a system function component, the finer its spatial structure. This behavior and the general spatial patterns closely resemble 2D Chebyshev polynomials, which can be written as a tensor product of the 1D polynomials for each direction: *U*_*n*_(*x*) ⊗ *U*_*m*_(*z*). Figure 9 plots the first components of these functions. The 2D Chebyshev functions satisfy an orthogonality relation similar to (50). A graphical representation of this relation for the first 256 components is shown in the left part of figure 10. The inner product between orthogonal functions vanishes, so that only the product of a function with itself is non-zero, leading to the diagonal line in figure 10. In the right part, the corresponding plot is shown for the normalized rows of an ideal particle 2D Lissajous system function. Bright spots and lines off the diagonal indicate that some system function components are not orthogonal with respect to each other. However, black regions prevail and one can infer that most components are orthogonal. Therefore, there is only little redundancy in the system function.

**Figure 11:**
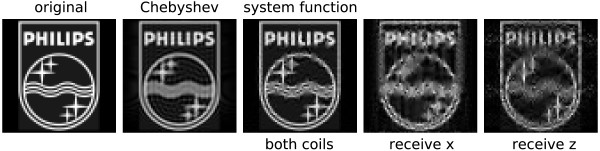
**Test Images**. 64 × 64 sample image and reconstruction from expansion into Chebyshev and system function components. The number of Chebyshev components and system function components using both coils was equal to the number image pixels. While the Chebyshev expansion represent only a hypothetical case shown for reference, the system function components can be derived from an idealized MPI experiment. Images on the right show the reconstruction result using only the system function components obtained from the receive coil in *x *and *z *direction, respectively.

To demonstrate this, a phantom image (figure 11, left) is expanded into an equal number of 2D Chebyshev and Lissajous system function components, respectively. The number of components has been chosen to equal the the number of pixels in the image (64 × 64). The image obtained from the Chebyshev transformation exhibits reduced resolution compared to the original image. The reason is that the Chebyshev functions provide higher resolution at the edges of the FOV but reduced resolution at the center. To keep the high resolution at the image center, higher Chebyshev components would have to be included in the expansion. The image obtained from the system function components has been reconstructed by inverting the system function matrix using minimal regularization to suppress noise [10]. Half the system function components were taken from the receive *x *system function, the other half from the *z *function (as displayed in figure 8). Resolution of the image is better than observed for the Chebyshev expansion, but the image has small artifacts that make it appear less homogeneous. Considering the fact that some system function components are not orthogonal and therefore redundant, the image quality is quite good. The reconstructions from only the *x *or *z *components show significantly worse image quality, indicating that these subsets are not sufficient to homogeneously represent the image information.

## Results and discussion

MPI signal encoding can provide a system function that forms a well-behaved basis set capable of representing highly resolved image information.

For 1D harmonic excitation of ideal particles, the system function corresponds to a series of Chebyshev polynomials of the second kind. Therefore, a fast and exact reconstruction is provided by the Chebyshev transform.

The properties of realistic particles are introduced into the system function by a convolution-type operation leading to a blurring of the high-resolution components. This introduces a resolution limit which is determined by the steepness of the particle magnetization curve. While in principle, a higher resolution image can be regained by deconvolution, resolution provided by realistic particles without deconvolution is already in the sub-millimeter range [3].

The system function for 2D imaging is determined by the trajectory taken by the FFP and a kernel representing the region around the FFP which contributes to the signal. The shape of this FFP kernel is determined by the topology of the selection field. The simple case of constant selection field gradients in all spatial directions has been demonstrated. For ideal particles, the kernel has sharp singularities which provide high spatial resolution. However, regions around these sharp peaks also contribute to the signal. This is probably the reason for the observation that in 2D encoding using a 2D Lissajous pattern, the system function is not exactly represented by 2D Chebyshev functions. Therefore, reconstruction cannot be done by using the Chebyshev transform as in 1D, but requires the inversion of the system function matrix. However, a close relationship between the 2D Lissajous system function and the 2D Chebyshev polynomials is obvious. This may be used to transform the system function into a sparser representation using a Chebyshev or cosine-type transformation, resulting in lower memory requirements and faster reconstruction.

The 2D Lissajous system function does not form a fully orthogonal set, since it contains redundant components. Nonetheless, it is capable of encoding highly resolved image information as shown in figure 11. Possible mismatches between the information content of the acquired data and the desired pixel resolution can be mediated by using regularization schemes in the reconstruction [10]. In experimentally acquired data, the necessary degree of regularization will also depend on the SNR. Optimally, to take into account noise in the system function as well as in the measured object data, image reconstruction should be based on the total least squares approach [11].

To speed up the tedious experimental acquisition of the system function, one can use the parity rules derived for the 2D system function. In theory, these allow to construct the complete system function from measuring only one quadrant of the rectangle of the Lissajous figure. For a 3D Lissajous figure, one octant would suffice, accelerating the system function acquisition by a factor of eight. Experimentally, the symmetry can be disturbed by non-perfect alignment of coils. Nonetheless, knowledge of the underlying theoretical functions and their parity can help to model the system function from only a few measured samples.

In a real MPI experiment, one usually acquires many more frequency components than the desired number of image pixels. Therefore, one has the freedom to make a selection of system function components to constitute a more compact basis set providing better orthogonality. For instance, duplicate system function components can be removed after acquisition to arrive at a smaller system function matrix to speed up image reconstruction. Furthermore, a selection of harmonics according to their weight can help to reduce matrix size. It is also conceivable to modify the weight of certain components to influence image resolution and SNR.

2D imaging of realistic particles has not been simulated in this work, but from the 1D derivations, one can infer that a blurring of the FFP kernel depending on the steepness of the particle magnetization curve will occur. This would remove the kernel singularities, but would also result in a slight loss of resolution, as discussed for the 1D case.

3D imaging has not been shown, but 2D results can be directly extrapolated to 3D by introducing an additional orthogonal drive field enabling 3D FFP trajectories. For a 3D Lissajous trajectory, close resemblance of the system function to third order tensor products of Chebyshev polynomials can be expected.

The selection field topology and the FFP trajectories used in this work have been chosen for their simple experimental feasibility. However, many alternative field configurations are conceivable. For the FFP trajectory, one can as well use radial or spiral patterns [12], or even patterns that are tailored to the anatomy to be imaged. Trajectories can be adapted to deliver varying resolution over the image. For the selection field, a topology creating a field-free line instead of the FFP promises more efficient scanning [8]. More research is required to identify field configurations optimal for specific applications.

## Conclusion

This work for the first time reports on the properties of the MPI system function. It shows that MPI signal encoding using harmonic drive fields in combination with constant selection field gradients provides a system function capable of representing highly resolved image information in rather compact form. The close relation to Chebyshev polynomials of the second kind can be used to speed up system function acquisition by partially modeling it, or to reduce memory requirements by applying tailored sparsity transforms resulting in faster reconstruction times.

The system functions explored here are tied to specific field configurations and scanning trajectories. Many other configurations are feasible, providing the flexibility to tailor system functions to satisfy certain experimental needs regarding speed, resolution, and sensitivity.

## Competing interests

All authors are employees of the Philips Technologie GmbH Forschungslaboratorien, Hamburg, Germany.

## Authors' contributions

JR and JW derived the mathematical treatment. JR furthermore wrote the manuscript, made the simulations and figures. BG and JB contributed ideas central to spatial encoding and image reconstruction in MPI and supported the development of the manuscript in helpful discussions. All authors read and approved the final version of the manuscript.

## Appendix

### A Mathematical Derivations for 1D Encoding

#### A.1 Signal Fourier Components Generated by a Periodic Drive Field

As shown in equation (3), the MPI signal is generated by the magnetization of particles following an externally applied (1D) field *H*. For spatial encoding, the field is split into a static selection field *H*_S_(*x*) which varies in space, and a homogeneous drive field *H*_D_(*t*) with a temporal variation, as shown in equation (6). The drive field is assumed to be periodic with frequency *ω*_0 _= 2*π*/*T*. Since the drive field causes a periodic change in magnetization, the acquired signal can be represented by a Fourier series(35)

Using *ω*_0 _= 2*π*/*T*, the coefficients are defined as(36)

To get rid of the time variable, parametrization is changed to the drive field by taking the inverse function of *H*_D_(*t*),

(37) *t *= *t*(*H*_D_),

which may require piecewise definition if the inversion is ambiguous. Ignoring this for the moment, equation (36) formally becomes(38)

where d*H*/d*t *= d*H*_D_/d*t *has been used.

In the following, (38) is evaluated making different assumptions on the type of drive field, selection field, and magnetization curve.

#### A.2 Fourier Components Generated by a Harmonic Drive Field

A harmonic drive field is easy to implement experimentally. With amplitude *A *and frequency *ω*_0_, one can write:

(39) *H*(*x*, *t*) = *H*_S_(*x*) - *H*_D_(*t*) = *H*_S_(*x*) - *A *cos *ω*_0_*t*.

**Figure 12:**
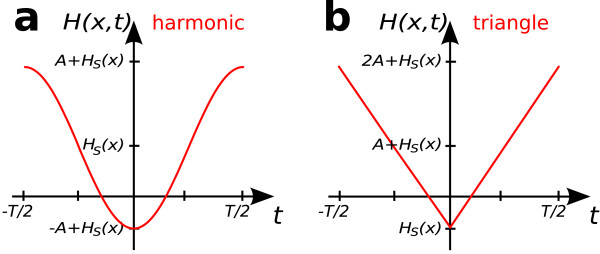
**Superposition of Drive and Selection Fields**. Superposition of selection and drive field at a spatial position *x*. The magnetization flips when the field *H*(*x*, *t*) = *H*_S_(*x*) - *H*_D_(*t*) crosses zero. The selection field offset *H*_S_(*x*) causes a time shift of the magnetization flip. Harmonic (a) and triangular (b) drive fields with amplitude *A *are displayed. Note that for mathematical convenience, the harmonic drive field ranges from -*A *to *A*, whereas the triangular field varies between 0 and 2*A*.

The cosine function has been chosen to arrive at a sine function after taking the derivative. The superposition of the two field components is shown in figure 12a. Time can be expressed as(40)

The two solutions have to be taken into account, and the integral (38) has to be split accordingly:(41)

Functions of the type sin(*n *arccos *x*) bear a close similarity to Chebyshev polynomials of the second kind [9]:(42)

Thus, (41) can be written as(43)

where  has been used. If we define(44)

the integration limits can be set to infinity. This is an artificial construction, since the drive field *H*_D _cannot reach infinite values, but it shows that (43) is a convolution:(45)

Thus, for a harmonic drive field, the frequency components form a set of Chebyshev polynomials, convolved with the derivative of the magnetization curve. Assuming a homogeneous drive field amplitude *A*, the spatial variation of the frequency components depends on the spatial variation of the selection field *H*_S_(*x*).

##### Addition of a Constant Field Gradient for 1D Spatial Encoding

To make an explicit choice for the selection field, a constant 1D field gradient *G *generating a field *H*_S_(*x*) = *Gx *is introduced. Then the frequency components have the following spatial dependence:(46)

##### Ideal Particles

For ideal particles, the magnetization curve corresponds to the sign function, so that, introducing the saturation magnetization *M*_0_, the magnetization becomes(47)

The derivative is the delta distribution

(48) *M'*(*H*) = *M*_0 _2 *δ*(*H*).

The convolution integral (43) then reduces to(49)

when a constant selection field gradient *G *has been assumed. The Chebyshev polynomials form a complete basis set and satisfy the orthogonality relation [9](50)

Therefore, the frequency components  measured by MPI can be used to represent any spatial particle distribution in the range -*A*/*G *<* x *<*A*/*G *accessed by the drive field.

#### A.3 Fourier Components Generated by a Triangular Drive Field

If a triangular drive field is used instead of the cosine field, the superposition of selection and drive field becomes

(51) *H*(*x*, *t*) = *H*_S_(*x*) - *H*_D_(*t*) = *H*_S_(*x*) - *A *tri *ω*_0_*t*

as shown in figure 12b. Note that in order to keep the functional form simple in later calculations, tri *ω*_0_*t *varies between 0 and 2, i.e., the imaging range has been shifted to 0 <* x *< 2*A*/*G*. Time can then be expressed as(52)

and the resulting signal components are(53)

##### Addition of a Constant Field Gradient for 1D Spatial Encoding

For a selection field *H*_S_(*x*) = *Gx*, the spatial dependence becomes(54)

##### Ideal Particles

For ideal particles, the integral (53) reduces to(55)

when a constant selection field gradient is assumed.

#### A.4 Derivation of the Chebyshev Transform from the Sine Transform

A function *f*(*x*) that is defined on the interval [0, *π*] and is zero at the edges of the interval, i.e., *f*(0) = *f*(*π*) = 0, can be mirrored to form an odd function over [-*π*, *π*] and thus can be expanded into a sine series. The following relations hold for the coefficients *b*_*k *_[13]:(56)

Using the substitution *x *→ arccos(*x*), one can easily show that the expansion changes to(57)

or,(58)

Since the range [0, *π*] is mapped to [-1, 1] by the coordinate transform, there is no symmetry requirement for *f*(*x*).

### B Mathematical Derivations for 2D/3D Encoding

Using a pair of detection coils in *z *direction, the acquired signal is proportional to the time-variation of the *z *component of the magnetization. According to (3),(59)

i.e., the total differential has to be taken. The same holds for receive coil pairs in *x *and *y *direction, so that (59) can be written in vector form:(60)

It should be noted that *∂****M***/*∂H*_*k *_generally is a function of ***H***(***r***, *t*), which can be chosen as the sum of a static selection field ***H***_S_(***r***) and a homogeneous drive field ***H***_D_(*t*). Assuming a 3D drive field that has an overall periodicity with cycle duration *T *= 2*π*/*ω*_0_, all three spatial components can be expanded into Fourier series, so that similarly to (36)(61)

The sum has been pulled out of the integral under the assumption that all individual integrals exist.

#### B.1 1D Harmonic Drive Field in *z *Direction

If a periodic 1D drive field is chosen in *z *direction,(62)

then only the time derivative of the *H*_*z *_component is different from zero(63)

The derivations for 1D encoding still hold for this situation. According to (38), the frequency components generated by a periodic drive field can be formally written as(64)

If a harmonic drive field *H*_D_(*t*) = *A *cos *ω*_0_*t *is used, the frequency components are based on Chebyshev polynomials, as derived in (45),(65)

The convolution operation only involves the *z *component of the field, which is why *H*_*Sz *_appears in the argument of the Chebyshev function.

##### B.1.1 Ideal Particles

Assuming ideal particles, the magnetization follows the external field as described by(66)

The differentials for the three spatial components are:(67)

3D spatial encoding requires a 3D selection gradient. An easy way to set up a 3D gradient is to use a Helmholtz pair of coils supplied with opposite currents in the two coils, also called the Maxwell coil [14]. Near the symmetry center, the generated gradient field can be approximated linearly:(68)

Including the above homogeneous drive field along the *z *direction, the total field becomes(69)

The spatial dependence of the signal picked up in three spatial directions (65) then becomes(70)

where the convolution involves the *z *variable only.

### C List of Symbols

*A *  drive field amplitude

*C*(***r***)  (true) particle concentration at position ***r***

  particle concentration convolved with *M'*(***r***), reconstructed concentration

  Fourier transform of particle concentration *C*(*x*)

***c***  concentration vector

*G*  magnetic field gradient

***H ***  magnetic field

***H***_*D*_(*t*)  drive field (spatially homogeneous)

***H***_*r*_(***r***)  field produced by a receive coil

***H***_*S*_(***r***)  selection field (static)

*I*_0 _  unit current

*k*_B _  Boltzmann constant

*μ*_0 _  magnetic permeability of vacuum

*m *  single particle magnetic moment

*M*_0 _  saturation magnetization

*M' *  derivative of the particle magnetization curve with respect to the magnetic field: 

***M***(***r***, *t* )  magnetization (depending on space and time)

*M*_*x*_(***r***, *t*)  magnetization component detected by *x *receive coil

*ω*_0 _  drive field frequency *ω*_0 _= 2*π*/*T*

***r ***  spatial coordinate vector

*s*(*t*)  signal detected from a point-like sample

*S*_*n *_  signal spectrum derived from *s*(*t*) by discrete Fourier transformation

***σ***_*r*_(***r***)  receive coil sensitivity

***S ***  system function matrix

*t *  time

*T*  cycle duration of periodic drive field excitation

*T*  temperature

*U*_*n*_(*x*) *n*th  Chebyshev polynomial of the second kind

*V*(*t*)  induced voltage

*V*  signal induced by particle distribution *C*(***r***)

***v ***  signal vector

Δ*x *  achievable spatial resolution without deconvolution

*ξ*  Langevin function argument *ξ *= *μ*_0_*mH*/*k*_B_*T*

## Pre-publication history

The pre-publication history for this paper can be accessed here:

http://www.biomedcentral.com/1471-2342/9/4/prepub
